# The use of portfolio to support competency-based professional development of pharmacists in a Singapore tertiary hospital

**DOI:** 10.15694/mep.2017.000138

**Published:** 2017-08-02

**Authors:** Sei Keng Koh, Camilla Ming Lee Wong, Mei Ling Yee, Dujeepa D. Samarasekera, Mun Moon Lim

**Affiliations:** 1 Department of Pharmacy; 2 Allied Health Division; 3 Department of Pharmacy; 4 Yong Loo Lin School of Medicine

**Keywords:** Pharmacy practice, competency framework, portfolio, professional development

## Abstract

This article was migrated. The article was marked as recommended.

Competency frameworks is implemented to support continuing professional development in an academic medical centre, with the aims of establishing and retaining a competent pharmacist workforce; and is described using Kotter’s change management framework. The desire to provide a clear developmental route with defined criteria to identify and bridge competency gaps through systematic training of our pharmacists and meeting JCI requirements for documented continued competence gave impetus to the establishment of the General and Advanced Level Frameworks. To aid implementation, a series of roadshows were organized to communicate the vision to pharmacists and experts from the UK were invited to share experience and to “train-the-trainers”. Clinical groups were set up to provide learning platforms for the advanced pharmacist practitioners to coach the pharmacists. Competency assessment was conducted biyearly using workplace based assessment tools. Formative feedback was provided post-assessment and learning objectives and training plan for the next assessment cycle would be discussed. The implementation of competency frameworks provides an opportunity for pharmacists to identify competency gaps and plan their training and development to achieve higher standards of practice. The portfolio and competency-based developmental frameworks enable systematic approach to evaluate and facilitate performance management.

## Introduction

Having a competent workforce is an essential pre-requisite to ensure quality outcomes for all healthcare professions. The capacity to improve therapeutic outcomes, patients’ quality of life, scientific advancement and enhancement of public health imperatives are dependent on a foundation of competence and continued development of practice (International Pharmaceutical Federation [FIP], 2012). Competence is generally taken to mean that an individual possesses the required knowledge, skills and attributes, gained through academic qualifications and supplemented by subsequent practice and training, to successfully and consistently perform a specific function or task to a desired standard ( Pharmaceutical Society of Australia, 2010).

Entry-level competency in Singapore-trained pharmacists is imparted through a rigorous 4-year undergraduate program [Bachelor of Science Pharmacy (Honors)] at the National University of Singapore (NUS), followed by a year of pre-registration training in an accredited training institution under the supervision of a qualified preceptor who certifies that the competencies for fitness to practice (entry to practice) have been met, as mandated by the Singapore Pharmacy Council (SPC) ( Singapore Pharmacy Council [SPC], 2011). The SPC accredits the training institutions and mandates that pharmacists have the required minimal two years of working experience and attend a preceptor workshop organized by SPC in order to qualify as a preceptor.

Post registration, continuing professional development of the pharmacist usually takes the form of on-the-job training and Continuing Professional Education (CPE). Mandatory CPE was introduced by SPC to ensure continued development and competence of pharmacists. Following revisions to the Pharmacists Registration Act in 2007, pharmacists need to achieve 50 CPE points over a 2-year period as a pre-requisite for renewal of their annual practicing certificate. A survey to measure pharmacists’ perceptions and attitudes toward mandatory CPE and perceived difficulty in fulfilling CPE requirements was conducted in 2013 ( Ang HG, Pua YH, Azah NA, 2013). The majority of pharmacists who responded indicated that mandatory CPE enhanced or increased their knowledge base and skills; and motivated them to continually learn as well as to reflect on their professional practice. However, they perceived that mandatory CPE does not enhance or increase employability and non-practicing pharmacists appeared to have the greatest difficulty in meeting the CPE requirements. As of May 2017, SPC has yet to implement a continuing professional development program which ensures that pharmacists are kept up-to-date on both their knowledge and skills.

Organizations engage in continuing or in-service competency assessments for a variety of reasons including meeting accreditation requirements as well as to assess for career development, career progression or merits ( Wright, 2005, pg1-7). Professionally, the ability to assess the knowledge, skills, attitudes, behaviours and specific competencies that underpin practice beyond entry-level is essential for providing safe patient care, and forms the basis of a professional development framework to support practitioner development for effective and sustained performance ( Royal Pharmaceutical Society (RPS), 2013). Over the years, developmental frameworks have gained recognition and popularity to act as tools to support pharmacists to ensure currency and for continued advancement in their practice.

This article describes the implementation of portfolio-based competency frameworks at the Singapore General Hospital’s Department of Pharmacy, the vision of which is to achieve positive patient outcomes through service excellence and optimization of drug therapy. The frameworks served to support continuing professional development, with the aim of establishing and retaining a competent workforce. We have organized the discussion using the change-management framework laid out by Kotter and colleagues which describes the key processes needed to conceptualize and execute transformational change within a large organization ( Kotter & Cohen, 2002; Kotter, 2007).

## Developing the framework

### Establishing a sense of urgency

The role and contribution of pharmacists to the overall healthcare system was evolving then - shifting from being product-centric (focusing on the safe distribution of pharmaceutical products) to being patient-centric, where pharmacists assumed greater responsibility for the practice and science of rational medication use ( White, 2006). Pharmacists who embraced this role and developed their clinical practice became increasingly frustrated with the lack of opportunities and management support for career progression in their clinical areas, and the department faced double digit attrition rates and negative feedback from its staff. This coupled with the need to compete for and retain top talents, created an urgency to re-look at our career pathway.

To acknowledge the clinical role that our pharmacists were increasingly involved in and to allow for better career progression, the original single-track pharmacy career pathway was bifurcated into two tracks with the establishment of the clinical track in the early 2000s. The establishment of the new clinical track allowed emplacement of aspiring clinical pharmacists and their development into future clinical leaders and advanced practitioners. The clinical specialist sub-track was established with introduction of the SPC Register of Specialists in 2012 and was incorporated into the career pathway to formally recognize our specialist pharmacists.

**Figure 1.**
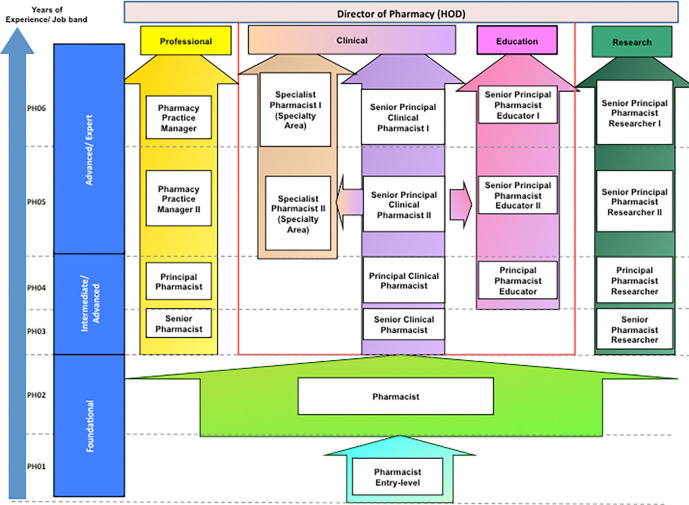
Career Development Pathway for Pharmacists in Singapore General Hospital

The rapid advancement in medical science and the transformation of SGH into an Academic Medical Centre prompted pharmacists to devote more time into research and education. Our pharmacists were actively involved in a number of areas of research including infectious diseases, ambulatory care, transplantation and hematology; and this acted as a catalyst for the establishment of the research track in the mid-2000’s. As the largest training institution for healthcare professionals in Singapore, our pharmacists are actively involved in the training and education of undergraduates and postgraduates (for pharmacists and other healthcare professionals). The career development pathway (CDP) underwent revision in 2014 to include the pharmacist clinical educator sub-track for aspiring clinical educators ( Figure 1).

The implementation of this CDP helped to define and aid career progression through articulation of requisites for emplacement onto the respective tracks, as well as promotion to the next higher job grade. Considerations for promotion include having the required years of experience, relevant professional post-graduate qualifications, certifications and demonstration of competence. In the desire to do the latter well, competency frameworks were developed and they helped to identify gaps in the knowledge and skills of our pharmacists, which in turn led to identifying the necessary education and training to bridge them.

### The quest and formation of a guiding coalition

The London-based Competency Development and Evaluation Group (CoDEG) is a collaborative network of specialist and academic pharmacists, developers, researchers and practitioners which was formed in 2001. Its objective is to undertake research to help develop and support pharmacy practitioners to ensure fitness to practice at all levels and to evaluate mechanisms to move the practice forward ( Competency Development and Evaluation Group [CoDEG], 2017). One of the chief accomplishments of CoDEG is the establishment of the evidence-based General Level Framework (GLF) and Advanced Level Framework (ALF) to support the development of pharmacists from licensure to general level practice and subsequently to advanced level practice ( Robbie, Webb, Bates, Wright & Davies, 2001; Meadows et al. 2004).

Our team of pharmacists reviewed CoDEG’s frameworks and supporting tools for their relevance and application in our institution. In addition, the content and structure of the GLF were analyzed and compared to the standards covered within the nation’s entry-to-practice framework. The team noted that the GLF was a developmental tool that could be used post registration and that encompassed the holistic scope of pharmacy practice with the aim of producing pharmacists who could provide safe and effective healthcare services. It was recognised that the GLF could also help to identify specific elements of practice for positive reinforcement as well as specific areas for development in a systematic approach. Regular use of the GLF had been shown to improve impact on patient care, job satisfaction and professional development ( Coombes I et al. 2010; Millis, Farmer, Bates, Davies, Webb, Robbie, 2005; Millis, Farmer, Bates, Davis, Webb,2008; Meštrovic et al. 2012). The competency domains in the ALF were also found to be applicable across the CDP. The three performance levels of each ALF standard described as Foundation, Excellent and Mastery could be used to define the competencies required to progress to the next higher level of practice. The decision was thus made to adapt the GLF and ALF for use in the Pharmacy Department at SGH.

In 2007, SGH collaborated with CoDEG and adaptation of the frameworks was done by a group of senior pharmacists actively practised in clinical setting and involved in education. The group reviewed each competency standard in the respective frameworks to ensure relevancy in the local context. The competency standards in the GLF were further revised to ensure that they built on the standards specified in the entry-to-practice competency framework. Through a mapping exercise, the workgroup also articulated the competency level required of pharmacists at each level of practice and across the various tracks. It was envisioned that the GLF would be used to support the development of post-registration, general level practice pharmacists for the first three to four years of their career, whereas the ALF would be used for advanced level practicing pharmacists after attaining competencies specified in the GLF.

Strong leadership is necessary to support transformation and its associated activities and with endorsement from hospital senior management, the pharmacy department invited key CoDEG leadership to Singapore to better understand the concepts and processes and to run a 5-days “train-the-trainer” workshop. During the training workshop, CoDEG shared with participants use of the GLF as an development tool and findings from pilot runs of the GLF assessments and a survey, which helped to identify competency gaps and subsequent fine-tuning of the pharmacists’ education and training ( CoDEG, 2009; Duncan, 2009; Coombes & Cardiff, 2009; Cardiff, 2009; Wong, 2009a). Intensive one-to-one ‘train the trainers’ sessions were conducted for 16 ‘super trainers’ who were carefully selected from the various SingHealth institutions. These ‘super trainers’ were trained on the practical application of the GLF and were expected to take lead in training GLF leaders in their respective institutions. A milestone in the continued collaboration between SingHealth and CoDEG was achieved in December 2009 when SingHealth signed a Memorandum of Understanding with CoDEG to adapt and use their GLF and ALF within the cluster. All SingHealth pharmacists were invited to an engagement dinner hosted by the cluster CEO to learn how the new CDP and competency frameworks were being implemented across the cluster and how they would benefit them ( Wong, 2009b). Between May and August 2009, the SGH version of the GLF was rolled out to 48 pharmacists and in 2010 the ALF was formally extended to all senior pharmacists in SGH.

### Creating and communicating a vision of competency-based development

An effective vision is described as imaginable, desirable, feasible, focused, flexible and communicable ( Kotter, 2007; Kotter & Cohen, 2002), and these are needed to inspire and mobilize any organization towards implementation.

In line with SGH efforts to establish an academic practice in our tertiary acute-care setting, we focused on strengthening excellence in clinical practice, education and research. We envisaged a practice where pharmacists were motivated through a well-defined career development pathway which provides avenues for personal career development, supported by competency frameworks. We articulated a vision where pharmacists will be developed in clinical practice in a structured training environment through competency-based development, with ample opportunities for learning and growth. They would be supported by a core group of advanced pharmacist practitioners, actively participating in education and research, who acted as coaches and mentors.

The workgroup spent considerable time soliciting buy-in and acceptance from pharmacists and their supervisors on use of the GLF and ALF to support competency-based development. Numerous engagement sessions were conducted in the presence of department management where concerns and criticisms were addressed. Pharmacists were taught how to use the frameworks and given tips on how to build an evidence portfolio to support their competency level. One-to-one coaching was provided to those who had difficulties building their portfolio.

### Removing obstacles and empowering others to act

#### Training program to support development of GLF pharmacists

The GLF consists of 72 behavioural descriptors covering three competency clusters in the areas of patient care delivery, problem-solving and professionalism ( Table 1). Use of the framework had to be supported by training programs to allow pharmacists to bridge the competency gaps identified. In conjunction with implementation of GLF, clinical groups (CGs) were formed and advanced pharmacist practitioners were identified to lead the CGs. The CGs served as a learning platform for pharmacists and ALF pharmacists to be coached and mentored by the CG leaders. During CG meetings, pharmacists discussed the latest advances in practice and clinical guidelines through journal clubs (journal evaluation) and case presentations.

**Figure 2.**
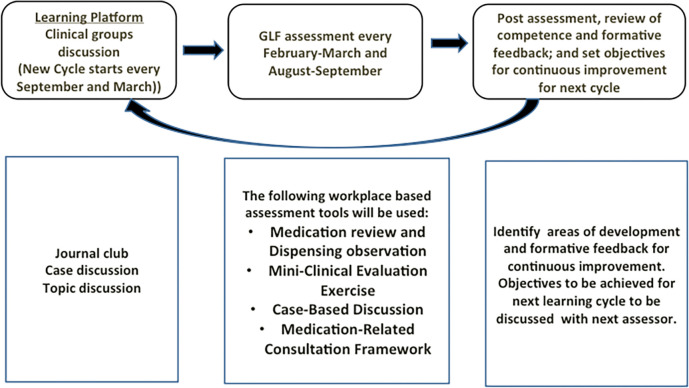
GLF Assessment Process and Learning Cycle

The competency of pharmacists were assessed by the CG leaders using Case-based Discussion (CBD) assessment, journal evaluation forms, direct observation of dispensing, medication review in ward and mini-clinical evaluation exercise (mini-CEXs). Formative feedback was provided with the aim for improvement in the next following presentation. The case presentations, intervention logs and multi-source feedback, if available, which formed the portfolio for pharmacist will be reviewed and assessed by the GLF assessors twice yearly. Based on the findings from assessment and portfolio, competency gaps would be identified and mutually agreed learning plans would be developed and thereafter, reviewed at the next assessment period which was 6 months later ( Figure 2). The CG leaders assumed an important role in this process to coach and bridge competency gaps so that the GLF pharmacists could be transited to the ALF upon attaining all of the stipulated GLF competencies.

### Development pathway for ALF pharmacists

The ALF is a portfolio-based framework, consisting of six competency domains and 34 competency standards ( Table 2). It is used in the annual assessment of senior level staff, i.e. pharmacists met GLF competency mapping and transited to ALF, by the respective immediate supervisors. The ALF portfolio is assessed at least annually by their appraisers, following which the goals of learning and development would be written down in the personal development plan (PDP). A department training roadmap to progress pharmacists from one practice level to the next level was subsequently developed to guide the learning needs of pharmacists. The training roadmap includes formal programs and workshops that pharmacists could attend to gain the necessary knowledge and skills. Experiential training is provided on-the-job and dependent on opportunities available at the institutional and cluster levels.

### Faculty development program and support

The department had to ensure adequate numbers of sufficiently skilled pharmacists to administer the frameworks and seamlessly implement them. Prior to implementation of the frameworks, pharmacists who were identified as assessors were sent for faculty development courses on providing effective feedback, coaching, facilitating skills, and the use of mini-CEXs and CBDs. The daily rosters were also revised to allow pharmacists to attend CG meetings which were initially held on a weekly basis. Following feedback that it was challenging to manage full operating patient loads with lesser numbers of pharmacists, the frequency was reduced to once every 2-4 weeks. Informal feedback was solicited periodically to gather inputs on use of the frameworks and assessment tools, and forms were updated following feedback from the pharmacists.

### Creating short term wins, building on change and institutionalize new approaches

It is suggested that sustaining change efforts requires compelling evidence of progress within 12-24 months ( Kotter, 2007). To evaluate the acceptability and validity of the GLF as a tool to facilitate and evaluate performance development for junior level pharmacists, observational evaluations during daily clinical activities were prospectively recorded for 35 pharmacists using the GLF at two time points over an average of 9 months ( Rutter et al. 2012). The key element of GLF was well demonstrated when feedback was provided to the pharmacists and individualized learning plans were then formulated and followed up. It was found that the pharmacists’ mean competency scores improved in all 3 clusters and significant improvement was seen in all but 8 of the 63 behavioral descriptors ( p<0.05). Feedback from pharmacists indicated that the GLF process was a positive experience which prompted reflection on practice and culminated into needs-based learning, which ultimately improved patient care.

A survey was conducted to obtain feedback on how satisfied the inpatient pharmacists were with the GLF process and how it had impacted their development and the results of GLF pharmacists and clinical group (CG) leaders were favorable. Although more than 90% of the pharmacists and CG leaders recognized the value of GLF on their development, only 55 % of CG leaders felt that GLF assessment reflected the pharmacists’ knowledge and skills. Both GLF pharmacists and CG leaders felt that protected time was required for CG presentations and assessments ( Yee & Wong, 2011).

The ALF was administered annually in April during the appraisal period from 2010 to 2013. Within each of the 6 domains, pharmacists self-reported competency level(s) for the respective sub domains, substantiated with portfolios. The competency development in our senior-level pharmacists through the utilization of the ALF over the 4-year period was studied. Out of 33 senior-level pharmacists assessed in 2010 and who were still employed in SGH at the time of study, data from only 22 (67%) could be used as 2 (6%) and 5 (15%) were on maternity and training leave respectively at time of appraisal, and the ALF was not administered to 4 (12%) pharmacists during the appraisal periods. Overall, 86% of staff demonstrated an increase in their competence, mainly in the areas of Expert Professional Practice (55%), Building Working Relationships (45%) and Leadership (45%). Over the 4 years of assessment, the domain in which most pharmacists showed improvement in was Expert Professional Practice. Building Working Relationships was the domain that pharmacists achieved Mastery level fastest, whereas Research was the domain that pharmacists showed least improvement in ( Wong & Lim, 2014).

## Discussion

### Reflections

The competency frameworks are in the 10th year of use at the Department of Pharmacy in SGH and they have helped to identify competency gaps in its staff and spurred the department on to bridge these gaps through formal education and work-based learning. In the last 2-3 years, we had also leveraged on the competency frameworks to identify suitable candidates for admission into our pharmacy residency programs such as Infectious Diseases and Critical Care.

### Factors influencing the successful use of portfolio to support CPD

Van Tartwijk describes a model of factors influencing the successful introduction of portfolio in education: people (teachers and learners), academic leadership and infrastructure ( van Tartwijk et al, 2007). Developing a portfolio and assessing it involves putting a lot of effort and time both from the assessor and the pharmacist. Self-assessment and reflection which are fundamental to portfolio building are not intuitive and at times challenging. We struggled in the beginning, having difficulties interpreting and identifying evidence to support achievement of competence and learning experiences to bridge competency gaps. Engagement and teaching sessions on use of portfolios helped to ease the struggle. Buy-in by stakeholders and support from the management is important and need to be sought early. Processes have to be reviewed to ensure ease of implementation of the CGs and assessment process to support learning without adversely affecting the delivery of patient care and medication supply. Mentoring and coaching by senior pharmacists also contribute to the success of the portfolio based competency framework. Our senior pharmacists invest much of their time to stimulate the development of critical thinking and clinical reasoning in the junior pharmacists and identify learning experiences for them to sharpen and hone their skills.

### Portfolio as developmental and assessment tool

In our department, the portfolio serves not only as a formative but a summative tool. Pharmacists have to meet the stipulated competency requirements and the years of experience before consideration for a promotion to the next job grade. However, it should be noted that promotion is not solely dependent on meeting the required competency but it is also an achievement of other attributes and meeting the core values of the institution.

In a survey conducted in 2015 on the usefulness of the ALF as a developmental cum assessment tool, 70% of the pharmacists (n=50) agreed that ALF is a useful tool for development and only 44% agreed that it is an objective assessment tool. This could reflect the need for robust training programmes for the assessors so that assessments are more objective and there is less variation between assessors.

### Limitation of the competency framework

The demonstration of competence for GLF involved the use of formative work-based assessment tools like direct observation of dispensing skills and Mini-CEX. A number of observations, multi-source feedback (8-12 peers) and mini-CEXs (about 12-14) have to be repeated to ensure reliability. ^27^ However because of time constraint , the number of dispensing observations and mini-CEXs is about 3-5 and there are occasions of competence lapses.

On the limitation of the ALF, pharmacists on the professional track have informed that the competencies in the Expert Professional Practice domain are geared towards direct patient care in the clinical areas and not relevant to those in indirect patient care e.g. IT or purchasing. Pharmacists have also commented that the ALF could be further individualized to cater to the needs of the individual as one size does not fit all. In addition, concerns were raised on the limited opportunities to identify the scope of practice at institutional or national level in order to develop the pharmacists to the highest level of competence and practice at the top of the license.

### Our next leap

Competencies are dynamic and continual review is needed to ensure its relevance. Surveys have been conducted to seek inputs on the challenges and issues faced in using the GLF. Several points have been raised by pharmacists, GLF assessors and CG leaders, including difficulties in identifying learning experiences required for bridging the competency gaps, and inter-assessor variability in assessment. Furthermore, challenges in giving effective feedback were highlighted and assessors had also expressed the desire to receive more training on assessment methods.

A competency workgroup has been identified to lead in the refreshment of the GLF and tools, and improve the process of administering the framework. The GLF handbook was last revised in 2013 to include more relevant competencies in local pharmacy practice and will be revised again with more emphasis on providing the knowledge, skills and attributes for each competency standard and help users identify the learning experiences needed to bridge specific competency gaps. The ALF is currently being revised and harmonized at the national level for all public institutions and this is targeted to be implemented by the end of 2017. In addition, the work done by pharmacy has influenced other Allied health professionals within the SingHealth cluster to revise their CDPs and develop similar competency frameworks.

Plans are also underway to identify and develop in-house or on-the-job education and training programs to support specific competency standards and moving forward, the department hopes to assess the impact of implementing such competency frameworks on patient outcomes.

## Conclusion

The competency frameworks are useful tools to evaluate and facilitate performance development in our pharmacists. They have helped pharmacists identify competency gaps and the necessary education and training needed to attain higher standards of practice. Our experience of using the CoDEG competency frameworks seem to indicate that they are flexible and applicable to this region, and can be customized to meet our local institutions’ needs and expectations.

## Take Home Messages

• Competency framework can help pharmacists to identify specific competency gaps and take steps to plan for their training and development thus promoting self-directed learning to achieve higher standards of practice.
• Competency frameworks can also be used for performance management. Coupled with a training roadmap, they can help to facilitate career progression.
• Supportive leadership and pharmacists’ buy-in are important for the successful implementation of competency frameworks, Competency standards are dynamic and have to be reviewed periodically to ensure relevance.

## Notes On Contributors

Sei Keng Koh, M. Clin Pharm, BCPS, is a Principal Clinical Pharmacist in the Department of Pharmacy, Singapore General Hospital and a Senior Pharmacy Administrator at the Chief Pharmacist’s Office, Ministry of Health Singapore. Her areas of interest include anticoagulation, assessment of learning and professional development.

Camilla Ming Lee Wong, Pharm D, is Director, Allied Health, Sengkang Health. She spearheaded the implementation of pharmacy competency frameworks in Singapore General Hospital and is leading implementation of the national pharmacy advanced-level competency framework, and development of career pathways and competency frameworks for 26 allied health professionals within the SingHealth cluster.

Mei Ling Yee, B Pharm (Hons), M. Clin Pharmacy, BCPS, is a Senior Principal Clinical Pharmacist overseeing the pharmacy clinical services in the Department of Pharmacy, Singapore General Hospital. A liver transplant pharmacist by training, she co-spearheaded the implementation of competency frameworks in the Department of Pharmacy, Singapore General Hospital.

Dujeepa D. Samarasekara, MBBS (Col.), MHPE(Maast.), FAMS(Sing), FAcdMEd(UK), FAMEE is the Director, Centre for Medical Education, Yong Loo Lin School of Medicine, National University of Singapore. His research interests are effective teaching/learning behaviours and assessment. He serves on editorial advisory boards and is faculty to local and international health professional education programs.

Mun Moon Lim, BSc (Pharm) (Hons), is Director, Pharmacy, Singapore General Hospital. He has advanced pharmacists’ roles in clinical practice, education and research had through re-structuring of the career pathways and establishment of competency frameworks. He reviews and endorses all departmental IRB applications for research studies and precepts in the Pharmacy Practice Residency Programme.

## Appendices

**Table 1.** Standards in the General Level Framework

Domain 1	Delivery of Patient Care Competencies
Patient consultation	
1.1	Opening the consultation
1.2	Questioning
Gathering information	
1.3	Allergies
1.4	Relevant patient background
1.5	Medication Reconciliation
Provision of medication	
1.6	Prescription is ambiguous
1.7	Prescription is legal
1.8	Labelling of the medicine
1.9	Medication supply
Drug specific issues	
1.10	Drug selection
1.11	Selection of formulation, concentration, rate and diluent
1.12	Checking of dose, frequency, timing, route and duration
1.13	Drug Storage
Patient Education	
1.14	Patient is counselled on medication
1.15	Compliance assessment
1.16	Need for information identified
1.17	Risk management
1.18	Service improvement
**Domain 2**	**Problem Solving Competencies**
2.1	Identification of drug-related problems
2.2	Prioritization
2.3	Intervention
2.4	Consultation or referral
Knowledge	
2.5	Pathophysiology
2.6	Pharmacology
2.7	Side effects and monitoring
2.8	Interactions (drug/disease/special interest groups)
Analysis and Recommendations	
2.9	Use of guidelines and evidence
2.10	Information provision to other healthcare professionals
2.11	Documentation of drug-related problems
2.12	Monitoring and problem resolution
**Domain 3**	**Professional Competencies**
Organisation	
3.1	Prioritization
3.2	Punctuality
3.3	Time management
3.4	Initiative
3.5	Task Management
Professionalism	
3.6	Professional code of ethics
3.7	Confidentiality
3.8	Confidence
3.9	Responsibility
3.10	Organisational
Communication Skills	
3.11	Communication
3.12	Staff development
3.13	Pharmacy team
3.14	Multidisciplinary team

**Table 2.** Standards in Advanced Level Framework

Domain 1	Expert Professional Practice
1.1	Expert Skills and Knowledge
1.2	Patient Care Responsibilities
1.3	Reasoning and Judgement
1.4	Professional Autonomy
**Domain 2**	**Building working relationships**
2.1	Communication
2.2	Teamwork and consultation
**Domain 3**	**Leadership**
3.1	Strategic Context
3.2	Clinical Governance
3.3	Vision
3.4	Innovation
3.5	Service development
3.6	Motivational
**Domain 4**	**Management**
4.1	Implementing National Priorities
4.2	Resource utilisation
4.3	Standards of Practice
4.4	Managing Risk
4.5	Managing Performance
4.6	Project Management
4.7	Managing change
4.8	Strategic Planning
4.9	Working across boundaries
**Domain 5**	**Education and training**
5.1	Role model
5.2	Mentorship
5.3	Conducting education and training
5.4	Continuing professional development
5.5	Links Practice and education
5.6	Education Policy
**Domain 6**	**Research and Evaluation**
6.1	Critical Evaluation
6.2	Identifies Gaps in the Evidence base
6.3	Develops and evaluates research protocols
6.4	Creates evidence
6.5	Research evidence into practice
6.6	Supervises others undertaking research
6.7	Establishes research partnerships

**Table 3.** Feedback on the usefulness of ALF as a developmental cum assessment tool

*The description in ALF is rather subjective. We need adequate examples to guide us in addressing our competency level. Different assessors may assess differently, hence the fairness and transparencies are not standardized.*
*The ALF is a very strenuous exercise and may not recognize other positive attributes and competencies of the staff.*
*Perhaps this competency framework is applicable to clinical skills. It should not applied wholesale to all pharmacy positions e.g management, research.*
*The ALF is extremely tedious and time consuming to complete. There is also a certain extent of subjectivity. Terminologies used (description of individual competencies) are vague and open to variable interpretation.*
*ALF is a good tool for assessing competency and identify knowledge gaps especially relevant in clinical or research setting. However, in my opinion, it should not be used solely for the need to promote a staff.*
*ALF helps to demonstrate the stage of advanced professional practice relevant to pharmacist’s role but should not be used as an assessment for job promotion.*
*The competency framework is still very subjective and some sections are irrelevant if a staff’s portfolio is very defined. And this affects the overall rating.*
*ALF sessions are rather interactive and learning experience; to a certain extent agree to the usefulness*
