# Supplementary material for: The use of portfolio to support competency-based professional development of pharmacists in a Singapore tertiary hospital
Source: MedEdPublish (2016). 2017 Aug 2;6:138. [Version 1] doi: 10.15694/mep.2017.000138 (PMC10885284; doi:10.15694/mep.2017.000138)
Supplement: Peer review history

# Reviewer report: Reviewer response for version 1

**DOI:** 10.21956/mep.19326.r28215

**Published:** 2017-08-03

**Authors:** Tan Nguyen

**Affiliations:** 1 Deakin University

This review has been migrated. The reviewer awarded 3 stars out of 5 The paper describes a practical application to improve competencies for pharmacists, which follows the growing need to move towards competency based medical education. There is probably some scope to add more detail on the outcomes where evaluation was conducted to support the findings. It was uncertain whether how the portfolios was maintained (eg. online, written, blended) or used, as there was little detail on the use of portfolios themselves. As previously mentioned, collecting feedback from consumers of pharmacy services would add value to the learning experience.

Reviewer Expertise:

NA

No decision status is available

# Reviewer report: Reviewer response for version 1

**DOI:** 10.21956/mep.19326.r28216

**Published:** 2017-08-02

**Authors:** Trevor Gibbs

**Affiliations:** 1 AMEE

This review has been migrated. The reviewer awarded 4 stars out of 5 A very interesting and well written paper, covering an important area in pharmacist development. I feel that this piece of work was very well thought through and realistically explained. I specifically like the approach taken of using a validated instrument and adapting it for the local environmentGiven the purpose of the activity, I would have liked to have seen more involvement in the users of the pharmacy services, i.e. employers and patients, in both the development and evaluation.I think that this approach is adaptable to other countries as well as to other health professions' education and would recommend it to those involved in CPD curricula development

Reviewer Expertise:

NA

No decision status is available
